# Cytotoxin- and Chemotaxis-Genes Cooperate to Promote Adhesion of *Photobacterium damselae* subsp. *damselae*

**DOI:** 10.3389/fmicb.2018.02996

**Published:** 2018-12-13

**Authors:** Gisela von Hoven, Claudia Neukirch, Martina Meyenburg, Sabine Schmidt, Ana Vences, Carlos R. Osorio, Matthias Husmann, Amable J. Rivas

**Affiliations:** ^1^Institute of Medical Microbiology and Hygiene, University Medical Center, Johannes Gutenberg University, Mainz, Germany; ^2^Departamento de Microbioloxìa e Parasitoloxìa, Instituto de Acuicultura, Universidade de Santiago de Compostela, Santiago de Compostela, Spain

**Keywords:** *Photobacterium damselae* subsp. *damselae*, pore forming toxin, phospholipase D, chemotaxis, *cheA*, swimming, salinity, adhesion

## Abstract

*Photobacterium damselae* subsp. *damselae* (*Pdd*) is an emerging pathogen of marine animals that sometimes causes serious infections in humans. Two related pore forming toxins, phobalysins P and C, and damselysin, a phospholipase D, confer strong virulence of *Pdd* in mice. Because infections by *Pdd* are typically caused following exposure of wounds to sea water we investigated how salinity impacts toxin activity, swimming, and association of *Pdd* with epithelial cells. These activities were low when bacteria were pre-cultured in media with 3.5% NaCl, the global average salinity of sea water. In contrast, lower salinity increased swimming of wild type *Pdd* peaking at 2% NaCl, hemolysis, and association with epithelial cells peaking at 1–1.5%. Previously, we have found that hemolysin genes enhance the association of *Pdd* with epithelial cells, but the underlying mechanisms have remained ill-defined. We here searched for potential links between hemolysin-production, chemotaxis and association of *Pdd* with target cells at varying salt concentrations. Unexpectedly, disruption of chemotaxis regulator *cheA* not only affected bacterial swimming and association with epithelial cells at intermediate to low salinity, but also reduced the production of plasmid-encoded phobalysin (PhlyP). The results thus reveal unforeseen links between chemotaxis regulators, a pore forming toxin and the association of a marine bacterium with target cells.

## Introduction

*Photobacterium damselae* subsp. *damselae* (*Pdd*), formerly *Vibrio damsela*, is a pathogen of marine animals (Rivas et al., 2013b; Osorio et al., 2018), which also causes septicemia and hyper-aggressive necrotizing soft tissue infections in humans (Clarridge and Zighelboim-Daum, 1985; Yamane et al., 2004). Hemolysins contribute to the virulence of *Pdd* for mice and fish (Rivas et al., 2013a). Strains of particular virulence produce plasmid-encoded damselysin (Dly) and phobalysin P (PhlyP), a phospholipase D, and a small pore-forming toxin (PFT), respectively. All hemolytic strains express chromosomally encoded phobalysin C (PhlyC), which is closely related to PhlyP. In addition, a phospholipase (PlpV) appears to be expressed by all strains; and a collagenase (ColP) is found in a subpopulation of *Pdd* (Vences et al., 2017; Osorio et al., 2018). *Pdd* is most likely transmitted by contaminated water (Fouz et al., 2000), and the most common entry ports for *Pdd* infections are wounds exposed to sea water (Morris et al., 1982; Rivas et al., 2013a). Bacterial adhesion to the host represents an important step to infection. Pili are established mediators of bacterial adhesion (Duguid et al., 1955, 1966; Solanki et al., 2018) including adhesion of *Pdd* (Rivas et al., 2015b). More recently, a role of hemolysins for the association of *Pdd* (Rivas et al., 2015b) or other bacteria (Krawczyk-Balska and Bielecki, 2005; Lucas et al., 2010; Vadia et al., 2011; Seitz et al., 2013) with host cells has emerged. However, the mechanisms have remained unclear.

Conceivably, directional movement toward host cells could increase the number of bacteria adhering to those cells. Chemotaxis enables flagellated bacteria to climb gradients of environmental stimuli to find niches of favorable conditions for their growth and survival (Berg, 1975; Adler, 1976; Colin and Sourjik, 2017). In the case of marine bacteria, chemotaxis is thought to facilitate utilization of nutrient patches in planctonic habitats (Blackburn et al., 1998). Similarly, chemotaxis could serve to facilitate colonization of a wound. In fact, there is evidence that chemotaxis and motility may influence bacterial colonization, infectivity or virulence in different ways (Butler and Camilli, 2004, 2005). Therefore, it is of interest to study the role of chemotaxis genes for the association of *Pdd* with host cells. The chemotaxis pathway of *Escherichia coli* has been thoroughly studied, and the basic architecture, composition and function of the chemotaxis apparatus are conserved, but certain aspects appear to be peculiar in marine bacteria (Son et al., 2016). The histidine kinase CheA has emerged as a conserved regulator of bacterial chemotaxis (Wadhams and Armitage, 2004). CheA is a component of the signal transduction module which transmits signals from trans-membrane chemoreceptors to flagellar motors. Although several studies have linked chemotaxis and adhesive properties in other experimental systems, information on the role of chemotaxis for the association of *Pdd* with mammalian cells is lacking.

In addition to factors intrinsic to bacteria or hosts, environmental parameters may influence the susceptibility to an infection (e.g., Reidl and Klose, 2002). In the case of marine bacteria salinity is naturally of specific interest. For instance, it has been shown that salinity impacts toxin secretion by *Vibrio vulnificus* (Lee et al., 2000), long-term survival of *V. vulnificus* biotype 2 (Marco-Noales et al., 1999), motility and chemotaxis of *V. anguillarum* (Larsen et al., 2004), and adhesion of *V. alginolyticus* (Huang et al., 2017). Also, *Pdd* hemolysin promoters were found to be more active when bacteria were cultured in 0.5% salt as compared to 3.5% salt (Rivas et al., 2013a). During infection of a mammalian host, halophilic organisms like *Pdd* experience a shift from a high salinity environment to a low salinity milieu.

We here asked whether PhlyP-dependent adhesion of *Pdd* (Rivas et al., 2015b) might depend on salinity and chemotactic motility. Therefore, we investigated the effect of NaCl concentrations on hemolysin production, swimming and the association of *Pdd* with human epithelial cells. To this end we used an array of *Pdd* strains which comprise deletions/disruptions in hemolysin genes and/or the single copy of the gene encoding chemotaxis regulator CheA (Table 1). The study led to the conclusion that a shift from high salinity to moderate salinity induces a transition of *Pdd* from an environmental mode to an infection mode. Surprisingly, this transition involves cross-talk of a membrane pore forming toxin and the chemotaxis apparatus, thereby enhancing adhesion to and damage of target cells.

**Table 1:** Bacterial strains used in this study.

Bacterial strain	Disruption in *cheA* gene	Reference	Toxins produced by the strains	Description
*P. damselae* subsp. *damselae*				
AR57	**-**	Rivas et al., 2011	PhlyP,PhlyC, Dly	RM-71 derivative, spontaneously rifampin-resistant mutant
AR267	**+**	This study	PhlyP,PhlyC, Dly	AR57 with disruption of *cheA* gene
AR281	**+**	This study	PhlyP,PhlyC, Dly	AR57 with disruption of *cheA* gene with reconstituted *cheA* gene
AR78	**-**	Rivas et al., 2011	PhlyC	AR57 with in-frame deletion of *hlyA_pl_* and *dly genes*
AR265	**+**	This study	PhlyC	AR57 with in-frame deletion of *hlyA_pl_, dly* and disruption of *cheA* genes
AR119	**-**	Rivas et al., 2013a	PhlyP	AR57 with in-frame deletion of *hlyA_ch_* and *dly* genes
AR267	**+**	This study	PhlyP	AR57 with in-frame deletion of *hlyA_ch_, dly* and disruption of *cheA* genes
AR275	**+**	This study	PhlyP	AR57 with in-frame deletion of *hlyA_ch_, dly* and disruption of *cheA* gene with reconstituted *cheA* gene
AR158	**-**	Rivas et al., 2013a	Dly	AR57 with in-frame deletion of *hlyA_pl_* and *hlyA_ch_*
AR266	**+**	This study	Dly	AR57 with in-frame deletion of *hlyA_pl,_ hlyA_ch_* and disruption of *cheA* genes
AR89	**-**	Rivas et al., 2013a	**-**	AR57 with in-frame deletion of *hlyA_pl_*, *hlyA_ch_* and *dly* genes
AR278	**+**	This study	**-**	AR57 with in-frame deletion of *hlyA_pl_*, *hlyA_ch_, dly* and disruption of *cheA* genes
*E. coli*				
S17-1 λ*pir*	**-**	Herrero et al., 1990	**-**	*recA thi pro* Δ*hsdR hsdM*^+^ RP4-2-Tc:Mu-Km:Tn7 λ*pir*; Tp^r^ Sm^r^

## Materials and Methods

### Bacterial Strains and Cultivation

*Photobacterium damselae* subsp. *damselae* (*Pdd*) RM-71 was isolated from diseased turbot during an outbreak in Galicia (Spain) (Fouz et al., 1992). A RM-71 rifampin-resistant derivative (AR57) was created (Rivas et al., 2011). AR57 and its Rif^R^ derivatives were routinely grown at 25°C on tryptic soy agar (TSA) (SIGMA 22091), or in trypic soy broth (TSB) (SIGMA 22092), with final NaCl concentrations of 1.5%. Sheep blood agar plates (Oxoid), containing tryptone, peptone, yeast extract, NaCl, pH 7.3 were used for conjugative mating. *E. coli* strains were routinely grown at 37°C in Luria-Bertani (LB) broth (ROTH) and LB agar [LB broth with 1.5% agarose (Agar-Agar Merck Millipore)], supplemented with antibiotics when appropriate. Antibiotics were used at the following final concentrations: kanamycin at 50 μg ml^-1^, ampicillin sodium salt at 100 μg ml^-1^, and rifampin at 50 μg ml^-1^.

### Mutant Construction and Phenotype Restoration

For construction of *cheA* mutants, *E. coli* S17-1 *λ*pir (Herrero et al., 1990) bearing the suicide plasmid pAJR80 (contains an internal fragment of 1734 bp of *cheA* gene) (Rivas et al., 2015b) was mated with diverse *Pdd* hemolysin mutant strains (see Table 1) as described in Rivas et al. (2011). Exponentially growing cells of donor and recipient strains were mixed, and 100 μl of the mix was placed directly onto a sheep blood agar plate, followed by incubation at 25°C for 2 days. Cells were scraped off the plate and re-suspended in TSB and 100 μl aliquots of serial decimal dilutions were spread onto TSA plates with the corresponding antibiotic combinations to select for donors and trans-conjugants. After conjugation, cointegration of the suicide vector into the chromosome by a single crossover results in *cheA* gene disruption and Km^R^ phenotype. Disruption of *cheA* gene was confirmed by polymerase chain reaction (PCR). Reversion of *cheA* mutants to a wild type *cheA* version was performed by growing *cheA* mutants in LB without kanamycin for five rounds to OD_600_ = 1.0 to facilitate suicide plasmid loss and subsequently selecting on LB with 15% sucrose for clones that have lost the cointegrate.

### DNA Manipulation and DNA Sequencing

Plasmid DNA or PCR-products were purified using NucleoSpin Plasmid Kit or NucleoSpin Gel and PCR Clean-up Kit according to the supplier’s protocol (Macherey-Nagel). Quality and quantity of DNA was assessed with a peQLab Nanodrop ND-1000 spectrophotometer. For verification of *cheA*-mutants long range PCR was performed with Qiagen HotStarTaq DNA polymerase using Thermocycler 2720 Applied Biosystems, and primer pair *cheA* check B and *sacB* check A (Table 2). Custom DNA Sanger sequencing was performed by StarSEQ GmbH.

**Table 2:** Primer used in this study.

Primer name	Sequence 5′→ 3′	Amplicon size (bp)
Primer for qPCR:		
*cheA* (1) primer A	**CCTGCTGCTGAAACTACTGTACGAGTTG**	319
*cheA* (1) primer B	**CCAGGTTCTTATCCAGATCGGTCTCTTCAC**	
*cheB* (1) primer A	**AAAGCCTCACATTACGGAGCACTCAGTACC**	267
*cheB* (1) primer B	**CCATCTTGCGCCTCTTTAACGCTGATCTTG**	
*cheZ* (1) primer A	**CCGCAATGGCAACGTTTGATGGATGGAG**	295
*cheZ* (1) primer B	**CACGCAATGCCCGTTCAACTTGTTGAGC**	
*hlyA*_pl_ primer A	**TGGTATCTGGTTGGGCTAGAGAATGGTC**	205
*hlyA*_pl_ primer B	**GCTTTCGGTCCATCTTTACTCACCTCTG**	
Primer for construction of *cheA* mutant:		
*cheA* primer A	**TCCTGAGAATTCCGAGTTAC**	1754
*cheA* primer B	**TACGCGTTCCTTTACCCAT**	
Primer for verification of *cheA* mutant:		
*cheA* primer check B	**AACCGATCCGTTGATGACCA**	2200
*sacB* primer check A	**AACCGATCCGTTGATGACCA**	

### Reverse Transcription-Quantitative Polymerase Chain Reaction

To determine whether disruption of *cheA* impacts the abundance of transcripts for *cheB, cheZ* and of hlyA_pl_, reverse transcription-quantitative polymerase chain reaction (RT-qPCR) was performed on cDNA samples derived from strains AR119 and AR267. Total RNA was isolated using the Trizol-Max Bacterial RNA Isolation Kit (Ambion), subsequent extraction with 1-Bromo-3 chloropane (Sigma), digestion with RQ1 RNAse-free DNAse (Promega). Quality and quantity of RNA was assessed with a Nanodrop spectrophotometer; and RNA integrity was verified with an Agilent 2100 Bioanalyzer instrument; RNA integrity number (RIN) ranged from 7 to 9. Reverse transcription of the purified RNA was performed with High capacity RNA to cDNA Kit according to the manufacturer’s protocol (Applied Biosystems). Subsequent qPCR was performed using Light Cycler 2.0 ROCHE and LightCycler Software Version build 4.1.1.21 (Idaho Technology, Inc.), Light Cycler Capillaries (20 μl) (ROCHE), LightCycler FastStart DNA Master SYBR Green I Kit (ROCHE) (includes SYBR Green Reaction Mix, FastStart Taq DNA polymerase, MgCl_2_) according to the kit protocols and specific primers listed in Table 2. qPCR-targets were *cheA* (>NZ_CP021151.1:789417-791720 amplicon size 319 bp), *cheB* (>NZ_CP021151.1:791795-793048 amplicon size 267 bp), *cheZ* (>NZ_CP021151.1:788685-789407 amplicon size 295 bp) and *hlyA*_pl_ (>NC_014653.1:c88749-86938 amplicon size 205 bp). Primer specificity was checked using BLAST/NIH. RT-qPCR-program: 40 cycles, denaturation 95°C 10 min; quantification at 95°C 10 s, 64°C 10 s, 72°C 18 s; melting curve 45°C 15 s; cooling 40°C 30 s. Amplification products were checked by analyzing melting characteristics and by electrophoresis on agarose gels. Cq at limit of detection (LOD) was between 32 and 34.5 for different targets. LODs were between 1 and 6 × 10^-7^ ng. Linear dynamic range was between 100–600 × 10^6^ copies (highest) and 30–130 copies (lowest) for different targets. Non-template-control (NTC) yielded negative results (NTCs Cqs ≥ 40). Number of technical replicates: *n* = 3.

### Bacterial Culture Fluids

Bacterial culture fluids (CFS) were obtained as follows. Bacteria were grown in LB until reaching exponential phase (i.e., OD_600_ = 0.4). Then bacteria were spun down and supernatants were sterilized by filtration through 0.2 μm filters. Protein concentrations in CFS were determined using a colorimetric protein assay (Bradford method) with Bio-Rad Protein Assay Dye Reagent according to the manufacturer’s protocol. CFS were stored for up to 24 months at -70°C without loss of activity.

### Motility Assays

Motility was generally measured by the use of a swim-migration assay (Adler et al., 1973). In this assay, bacteria migrate in response to a gradient of amino acids, created by their own metabolism. Overnight cultures were diluted to OD_600_ = 0.15 and 4 μl of each isolate was inoculated in the middle of a semi-solid LB plates containing 0.22% agar by vertically piercing the agar half-way and then gradually dispensing the culture while removing the pipette tip. Horizontal spreading of the culture was carefully avoided. Plates were incubated 24 h at 22°C. Growth diameters around the puncture sites were measured. To measure salt dependent spreading, adequate NaCl amounts were added during plate preparation.

A capillary assay format with identical buffer inside and outside the capillary was used to verify the loss of motility of *Pdd* in the presence of phenamil, an inhibitor of the flagellar motor, because swim-migration assays with phenamil in soft-agar did not yield robust results, possibly due to the interaction of the compound with components of the agar. The assay was basically performed as described previously (Adler, 1973; Larsen et al., 2004). In brief, disposable 5 μl pre-calibrated glass pipettes (Vitrex) were heat-sealed and filled with chemotaxis buffer (PBS with 0.8% NaCl and 0.01 mM EDTA). The open end of filled capillaries were immersed in bacterial suspension (freshly grown from OD_600_ = 0.1 to OD_600_ = 0.4) for 30 min at 22°C. Subsequently, capillaries were removed and the outside walls were briefly rinsed in chemotaxis buffer. The sealed end was broken and the content was emptied into a microfuge tube containing 1 μl of 0.9% NaCl. Serial dilutions of this suspension were made and 100 ml aliquots were spread on LB-plates. CFU were determined after 2 days incubation at 22°C. To quantify bacterial growth, overnight liquid cultures were adjusted to OD_600_ = 0.15 and diluted 1/14 (yielding an OD_600_ < 0.01 at *t* = 0) in LB with different salt concentrations. Samples were measured every hour for up to 5 h of incubation at 22°C and shaking (160 rpm).

### Hemolysis

Hemolysis assays were performed with RRCS [rabbit erythrocytes whole blood in Alsevers 1:2 (preclinics GmbH)]. The release of hemoglobin was measured in the supernatant at A_405_. Selected *Pdd* mutants were propagated in culture until they reached OD_600_ = 0.15 and 1/1000 dilution was diluted in original media containing salt concentrations ranging from 0.5 to 3.5% NaCl for an overnight incubation. Assays were carried out by mixing washed RRCs with supernatants from overnight cultures grown in salt concentrations ranging from 0.5 to 3.5% NaCl. Because high salt concentrations (3%) induce RRCs lysis, all supernatants were equilibrated to 2% NaCl which does not lead to cell lysis. 2% NaCl-balanced supernatants were serially twofold diluted in PBS in microtiter plates. 50 μl of diluted supernatant were added to 50 μl of 5% rabbit erythrocytes in each well and subsequently incubated at 37°C for 1 h. Absorbance of supernatants was measured at 405 nm with an ELISA reader. Maximum lysis (defined here as 100% lysis) was determined by resuspending pelleted red cells from 50 μl of 5% rabbit erythrocyte suspension in 100 μl *aqua dest*.(W4502 SIGMA); background lysis was determined in PBS.

### Phospholipase Assay

For measuring NaCl- or *cheA*-dependent phospholipase-activity filtered supernatants of *Pdd* were prepared as described below: one colony of each strain (AR158 and AR266; i.e., Dly-producing, with or w/o disruption of *cheA*), was freshly picked from a plate and grown in LB containing 0.5% NaCl to OD_600_ = 0.15 (AR266 with 50 μg/ml kanamycin). Afterward 5 μl of each strain was added to 6 ml LB of different NaCl concentrations (0.5–3.5%) and grown overnight (O/N) at 22°C. On the following day bacteria culture was spun down and the supernatant was filtered with a 0.2 μm Milipore syringe filter. Then the NaCl concentration was adjusted to 2% in all samples before 15 μl were filled into punched holes on egg yolk-agar-plates (Habermann and Hardt, 1972). After overnight incubation at 22°C diameters of halos due to phospholipase activity were determined. For preparation of egg yolk agar a mixture consisting of 15 ml LB-agar and 300 μl of a diluted fresh egg yolk (1:1 with LB) was warmed to 56°C and poured into plastic Petri dishes.

### Cells and Culture Conditions

HaCaT cells (RRID:CVCL_0038) (non-virally transformed human keratinocytes) (Boukamp et al., 1988) were cultured in DMEM/F-12 GlutaMAX^TM^-I medium (Gibco) with 10% fetal calf serum, 1% HEPES buffer (*N*-2-hydroxyethylpiperazine-*N*-2-ethane sulfonic acid) (Gibco), 1% penicillin/streptomycin (Gibco) in a humidified incubator with 5% CO_2_ at 37°C. All media and medium additives were obtained from Gibco by Life Technologies^TM^. Normal Human Epidermal Keratinocytes (NHEK) of an adult donor were purchased from PromoCell and cultured in Keratinocyte Media 2 with supplements as detailed by the supplier.

### Microscopy Based Adherence Assay

HaCaT cells (RRID:CVCL_0038) were seeded at a density of 1.5 × 10^5^ cells/well on glass coverslips and bacteria were grown overnight in LB containing 1.5% NaCl. The next day, bacteria were diluted 1/1000 in LB and propagated until reaching exponential phase (i.e., OD_600_ = 0.4). Bacteria were recovered by centrifugation (3200 *g*, 10 min at 4°C) and the pellet was re-suspended in cell culture medium (DMEM/F-12 GlutaMAX^TM^-I medium with 10% fetal calf serum, 1% HEPES buffer without antibiotics). Cells were washed twice with antibiotic-free cell culture medium to remove remains of antibiotics which were present during cell culture. Subsequently, bacterial suspensions were added to cells (MOI: 1:30) and the co-culture was incubated for 15 min at 37°C. Next, cells were washed twice and fixed with 2% paraformaldehyde in PBS for 10 min at 22°C. Nuclei and bacteria were stained with Hoechst 33342 (Cell Signaling Technology) and coverslips were mounted on slides with Fluoprep (bioMérieux SA). Samples were analyzed in a Zeiss Axiovert 200M epifluorescence microscope equipped with a Plan Apochromat 100x/1.4 numerical-aperture oil-immersion differential interference contrast (DIC) objective. For DIC microscopy, a Zeiss POL filter set was used. Digital images were acquired with a Zeiss axiocam camera. Image processing was done using Zeiss AxionVision software rel. 4.8., and Adobe Photoshop.

### Adhesion Assay Based on CFU-Counts

HaCaT cells (RRID:CVCL_0038) were seeded at a density of 2 × 10^5^ cells/well into six-well plates and bacteria were grown overnight in LB containing 1.5 or 3.5% NaCl at 22°C, vigorous shaking (160 rpm). The next day, bacteria were diluted 1/1000 in LB and propagated until reaching exponential phase (i.e., OD_600_ = 0.4). 100 μl/well were recovered by centrifugation (3200 *g*, 10 min at 4°C) and the pellet was re-suspended in cell culture medium (DMEM/F-12 GlutaMAX^TM^-I medium with 10% fetal calf serum, 1% HEPES buffer without antibiotics), cell culture medium with CFS obtained from *Pdd* that produce PhlyP, PhlyC, and Dly (wild type) or inhibitor (Table 3). Cells were washed twice with PBS to remove antibiotic included in cell culture medium. Subsequently, bacterial suspensions were added to cells and the co-culture was incubated for 15 min at 37°C. Next, cells were washed twice before they were harvested and re-suspended in 500 μl PBS. Dilutions of each sample were prepared, plated onto LB-agar and incubated overnight at 22°C. Finally, colony counts were assessed by two independent observers.

**Table 3:** Effect of small molecular weight inhibitors on the association of *Pdd* with HaCaT (*n* = 3).

Drug	Function	Strain	CFU absence/presence of drug	*p*	Significance
Dynasore 120 μM	Inhibitor of dynamin	AR 57	1.88	0.0001	***
Dynasore 120 μM	Inhibitor of dynamin	AR 89	1.18	0.3073	n.s.
Dynasore 120 μM	Inhibitor of dynamin	AR119	3.67	≤ 0.0001	***
EDTA 10 mM	Ion chelator	AR 57	2.13	0.0006	***
EDTA 10 mM	Ion chelator	AR 89	0.99	0.9283	n.s.
Cytochalasin D	Inhibitor of actin polymerization	AR 57	0.94	0.6588	n.s.
Cytochalasin D	Inhibitor of actin polymerization	AR 89	0.80	0.2462	n.s.
Nystatin 25 μg/ml	Induces of lipid raft	AR 57	1.28	0.0126	^∗∗^
SB203580 20 μM	Inhibitor of p38	AR 57	1.05	0.7660	n.s.
Ikarugamycin 4 μM	Inhibitor of clathrin-coated pit-mediated endocytosis	AR 57	1.07	0.4717	n.s.
Desipramine 30 μM	Inhibitor of acid sphingomyelinase	AR 57	1.17	0.4260	n.s.
BAPTA-AM 50 μM	Cell permeant ion chelator	AR 57	1.29	0.2077	n.s.
3-Methyladenine 5 mM	Inhibitor of type III PI-3K	AR 57	1.33	0.1994	n.s.
SR3576 20 μM	Inhibitor ofJNK3	AR 57	1.24	0.1702	n.s.

**p ≤ 0.01, ***p ≤ 0.001.

### Dot Blot

Filtered supernatants of *Pdd* cultures grown in LB containing 0.5% NaCl were adjusted to 2% NaCl before 200 μl of each sample were applied onto a nitrocellulose membrane (Whatman Protran Nitrocell transformed membrane). After blocking for 1 h at 22°C in skim milk (Milchpulver blotting-Grade ROTH) dissolved in Tris-buffered saline (TBST: 20 mM Tris pH 7.5, 150 mM NaCl, 0.05% [vol/vol] Tween 20), the membrane was incubated with a primary antibody directed against PhlyP (von Hoven et al., 2017), washed three times in TBST and incubated with horseradish peroxidase (HRP)-conjugated second antibody for 1 h at 22°C. Recombinant PhlyP (Rivas et al., 2015b) served as positive control. After three washing steps, bound antibody was detected by ECL (Roche Applied Science). Signals were quantified by densitometry using Image J (Rueden et al., 2017).

### Propidium Iodide Influx Assay

HaCaT cells (RRID:CVCL_0038) were seeded at a density of 4 × 10^5^ cells/well and bacteria were grown overnight in LB containing 1% NaCl at 22°C, shaking at 160 rpm. The next day, bacteria were diluted 1/1000 in LB and propagated until reaching an exponential phase (i.e., OD_600_ = 0.4). Bacteria were recovered by centrifugation (3200 *g*, 10 min at 4°C) and the pellet was re-suspended in PBS. Cells were washed twice with PBS to remove antibiotic included in cell medium. Subsequently, bacterial suspensions were added to cells (MOI: 1:30) and the co-culture was incubated for 15 min at 37°C. Next, cells were washed, detached, spun down and resuspended in PBS/EDTA 1 mM. After addition of propidium iodide (PI) (50 μg/ml), and incubation for 1 min cells were analyzed by flow cytometry using a FACScan instrument (BD).

### Statistical Analysis

Data displayed are derived from *n* ≥ 3 independent experiments, if not stated otherwise. Statistical significance of differences between two mean values was generally assessed with Student’s *t*-test; *p* ≤ 0.05 was considered to indicate statistical significance. Mann–Whitney test was used for non-parametric comparison of two mean values. A two-way ANOVA was employed to analyze RT-qPCR-data. For multiple comparisons, one-way ANOVA in conjunction with Tukey’s *post hoc* analysis was applied where appropriate. All assays were performed using GraphPad Prism 6 software.

## Results

### Swimming of *Pdd* Peaks at Intermediate Salt Concentrations and Is Inhibited by Disruption of *cheA*

**FIGURE 1:**
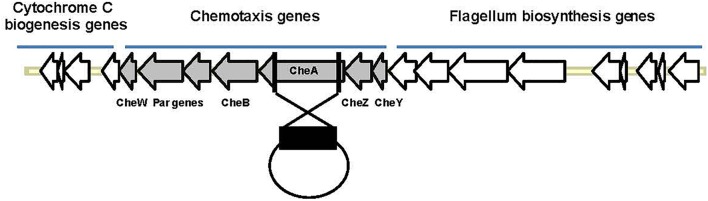
Organization of the chemotaxis gene cluster in *Pdd* and disruption of *cheA*. The schematic highlights the clustered organization of genes involved in chemotaxis or synthesis of flagella in *Pdd*. In order to disrupt chemotaxis, a large internal fragment of *cheA* (black rectangle), the largest che-gene within that cluster, was cloned into the suicide plasmid pNidKan which upon introduction into various *Pdd*-strains by conjugation recombines with, and disrupts, the single *cheA* gene of *Pdd*.

**FIGURE 2:**
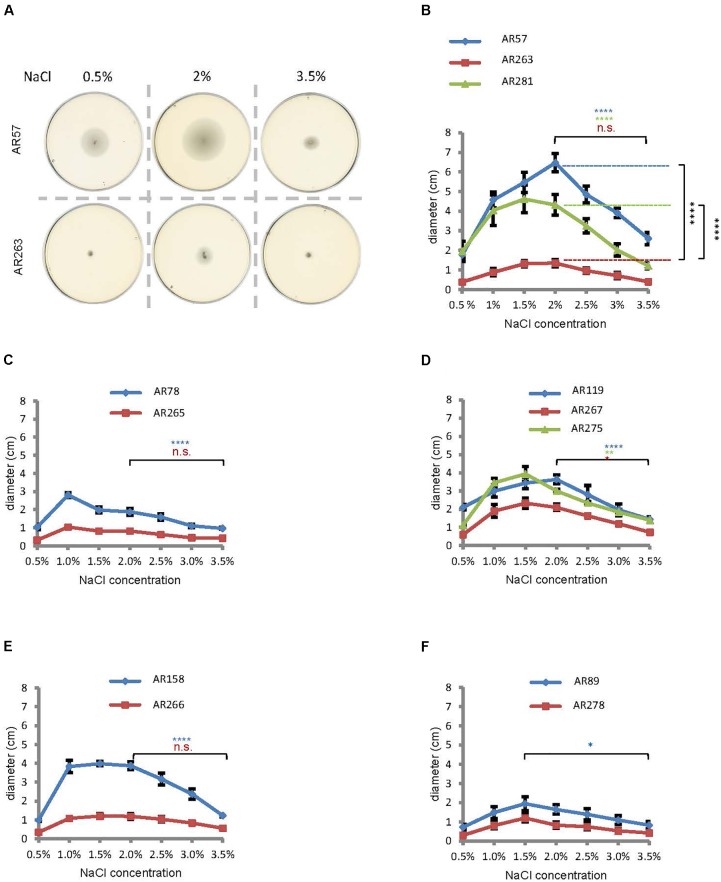
Salinity regulates swimming in *Pdd* and disruption of *cheA* impairs swimming. **(A)** Exemplaric *cheA-* and salt-dependent swimming patterns of *Pdd* on soft-agar-plates. **(B through F)** Summary of data for various *Pdd* strains (see Table 1). **(B)** *Pdd* that produce PhlyP PhlyC and Dly **(C)** *Pdd* that produce PhlyC, **(D)** *Pdd* that produce PhlyP, **(E)** *Pdd* that produce Dly, **(F)** *Pdd* with no hemolysin production and their respective *cheA* mutants (disruption of *cheA* or mutant with reconstituted *cheA* gene) were incubated on soft agar plates with indicated NaCl concentrations, and diameter of disk was measured after 24 h. Data shown are mean values ± SEM; *n* = 5. Four asterisks: (*p* ≤ 0.0001) two asterisks: (*p* ≤ 0.01) in a multiple comparison analysis using ANOVA with Tukey’s *post hoc* test; for comparisons between salt concentrations, color of asterisks matches the color of corresponding lines in graphs.

Salinity impacts the motility of *Vibrio* species (e.g., Larsen et al., 2004). To investigate whether motility of *Pdd* is regulated by salinity, we performed swimming assays on soft-agar with wild type *Pdd* strain (WT), AR57, at NaCl concentrations ranging from 0.5% w/v [i.e., lower than in serum (0.9%)] to 3.5% (the global average salinity of sea water). In order to verify that swimming of *Pdd* on soft-agar depends on its chemotactic ability, we studied a *Pdd* strain containing a disruption of its only *cheA* gene (AR263) (Rivas et al., 2015b). The chemotaxis cluster of *Pdd* and the disruption of *cheA* are depicted in Figure 1. The highest swimming activity of WT *Pdd* (AR57) was found at 2% NaCl, a concentration lower than in average sea water but higher than in serum (Figures 2A,B). The *cheA*-disruption-mutant (AR263) exhibited a >5-fold reduced ability to spread on soft-agar at intermediate salinity; and spreading was recovered in a reconverted strain (AR281) (Figure 2B). Soft-agar experiments were extended to include *Pdd* strains containing double deletions of two of the three major hemolysins (PhlyP, PhlyC and Dly) each, combined, or not, with a disruption of *cheA* (Figures 2C–E). In addition, strains AR89 and AR278 were studied, which are devoid of PhlyP, PhlyC and Dly, and in the case of AR278 of functional *cheA* (Figure 2F). In all strains containing deletions of toxin genes swimming was reduced to varying extent, but invariably, disruption of *cheA* led to additional decreases of swimming, which was most impressive with the double mutant strain expressing Dly alone (Figure 2E).

To sum, data from soft-agar assays indicated that swimming of *Pdd* peaks at 1–2% NaCl, and that disruption of *cheA* leads to significant reduction of swimming, in particular in the WT strain.

### Hemolysin Production by *Pdd* Peaks at Intermediate-to-Low Salinity

**FIGURE 3:**
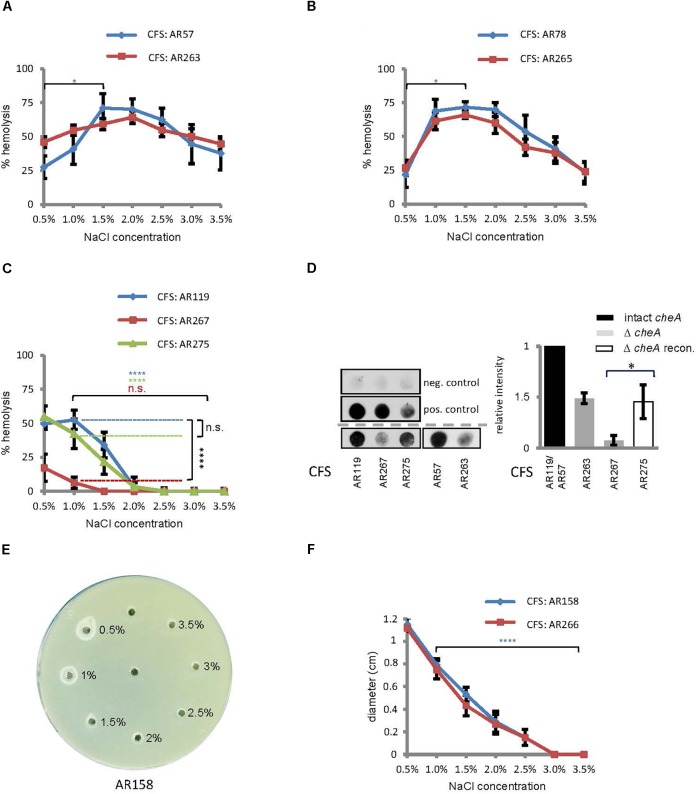
Salinity impacts hemolysin production and disruption of *cheA* reduces the production of PhlyP. **(A–C)** Culture fluids (CFS) of indicated *Pdd* strains grown in LB containing different salt concentrations were diluted and hemolytic activity was measured. Shown are mean values ± SEM (*n* = 5). **(D)** Dot-blot immuno-assay for PhlyP present in CFS (200 ml per dot). Left: representative blots upper panel, negative control (PBS); middle, positive control (from left to right: 6, 3, and 1.5 mg PhlyP); lower panels, CFS from indicated strains. Graph on the right hand: summary of data from *n* = 3 dot-blot experiments; mean ± SEM; asterisk indicates *p* ≤ 0.05 (Student’s *t*-test). **(E)** Phospholipase assay. 15 μl CSF, obtained at various salt concentrations and adjusted to 2% NaCl, were filled into holes punched into egg-yolk-agar plates. Plates were incubated overnight at 22°C. A representative plate is shown. **(F)** Summary of data obtained in experiments as shown in **(E)**; diameters of halos, corresponding to phospholipase activity, were determined after 16 h incubation of plates at 22°C. Shown are mean values ± SEM (*n* = 6).

Within a host, hemolysins will help to establish an infection by *Pdd*, but these toxins could be dispensable for much of *Pdd*’s planctonic life in a marine environment. Then it would be meaningful if hemolysin production was tightly controlled under environmental conditions, but up-regulated upon encounter with a host. To investigate whether a shift in salt concentration could serve as a cue to regulate hemolysin production, we measured hemolysis of rabbit red cells (RRCs) exposed to culture fluids from *Pdd*, grown at 0.5 to 3.5% NaCl. *Pdd* WT bacteria produced the highest hemolytic titers at intermediate salt concentrations (1.5%) (Figure 3A), thus resembling the pattern observed in motility assays, although the peak of hemolytic activity was slightly shifted toward lower salt concentrations. Hemolytic activity of a strain producing only PhlyC showed a similar bell shaped pattern of salt-dependency as the WT strain (Figure 3B). In contrast, lytic activity in culture fluids of *Pdd* strains expressing either of the two plasmid-encoded toxins (PhlyP, Dly) was basically inversely correlated with NaCl concentrations; in the case of PhlyP it was close to nil at ≥2% NaCl and reached a maximum at 1% (Figures 3C,F). Because RRCs are not lysed by Dly, a phospholipase assay was employed to measure Dly activity (Figures 3E,F). There was virtually no hemolysis of RRCs with culture fluids (CFS) of *Pdd* strains devoid of PhlyP, PhlyC and Dly, but complementation of AR89 with *hlyA*_pl_ reconstituted hemolysis (Supplementary Figure S1 and Supplementary Table S1).

These data supported the idea that salinity regulates hemolysin production in *Pdd*: generally, low to intermediate salinity promotes hemolysin production; and production of plasmid-encoded toxins tends to be particularly high in a more host-like environment, in regards to salinity.

### Disruption of *cheA* or Treatment With Phenamil Reduces the Production of PhlyP

The above results show that both swimming and hemolytic activity of *Pdd* is controlled by salinity. Conceivably, some proteins might regulate both functions. Therefore, we examined whether disruption of *cheA* would affect hemolytic titres in cultures of *Pdd*. Disruption of *cheA* in a WT background (regarding toxin genes) barely affected total hemolytic activity in CFS (Figure 3A). A strain only secreting chromosomally encoded phobalysin (PhlyC) yielded a similar pattern (Figure 3B). However, in the *Pdd* strain which expresses plasmid-encoded phobalysin (PhlyP) as the sole major hemolysin, disruption of *cheA* led to a strong reduction of hemolytic activity in culture fluids (Figure 3C). A dot-blot assay with an antibody against phobalysin, revealed that disruption of *cheA* markedly decreased the production of the toxin (Figure 3D). Reconversion of the *cheA*-disruption mutant partially restored toxin levels. To sum, disruption of *cheA* reduces the production of PhlyP (Figure 3D).

**FIGURE 4:**
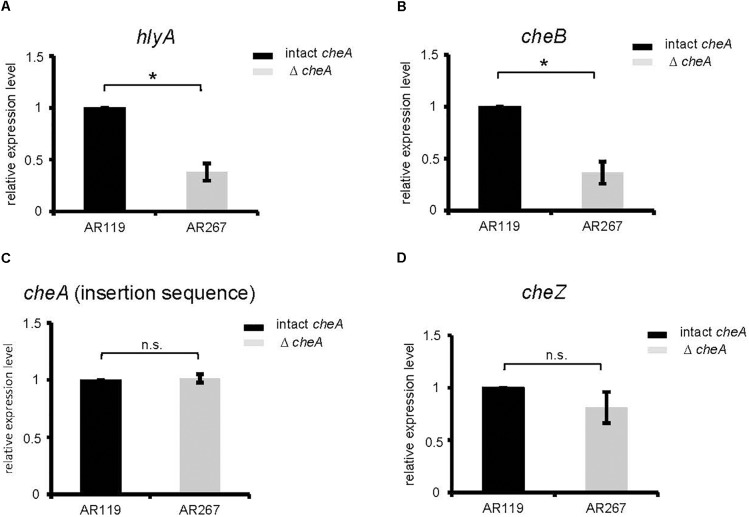
Disruption of *cheA* reduces PhlyP transcript levels. **(A–D)** Expression of *hlyA*_pl_, *cheB, cheA*, and *cheZ* was determined by RT-qPCR on cDNAs obtained from AR119 vs. AR267. Graphs display relative expression levels (normalized for AR119). Gray columns indicate mean values from *n* = 5 for *cheA, cheB*, *hlyA*_pl_, and *n* = 4 for *cheZ*; error bars indicate ± SEM. Asterisks highlight significant differences between mean values as determined by two way ANOVA on untransformed data, with experiment and strain as variables.

Therefore, we investigated whether disruption of *cheA* alters steady state levels of PhlyP transcripts. Indeed, RT-qPCR revealed that disruption of *cheA* in the background of AR119 (yielding strain AR267) leads to ∼2.5-fold reduction of *hlyA*_pl_ expression (Figure 4A). Also, expression of *cheB*, the gene located immediately downstream of *cheA* was reduced in AR267 (reference sequence NZ_CP021151.1 791795–793048 and 789417–791720, respectively) (Figure 4B). However, *cheZ*, which is located upstream of *cheA* (reference sequence NZ_CP021151.1 788685–789407 and 789417–791720, respectively), or the internal *cheA* sequence, which was used to disrupt that gene by insertion, were equally expressed in AR119 and AR267, and therefore serve as reference genes (Figures 4C–D). The collective RT-qPCR data indicated that disruption of *cheA* reduces steady state levels of PhlyP mRNA; and it is possible that down-regulation of *cheB* is involved.

**FIGURE 5:**
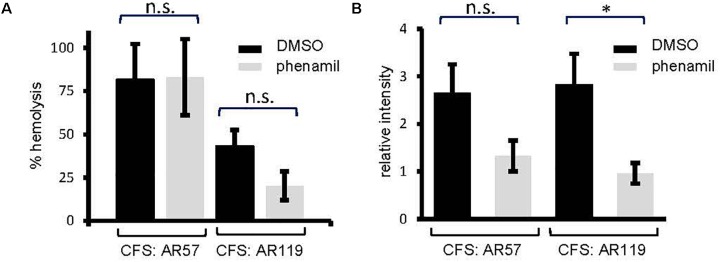
An inhibitor of the sodium-driven flagellar motor reduces the production of PhlyP (Figure 3D). **(A)** Culture fluids (CFS) of *Pdd* strains indicated in the legend were tested for total hemolytic activity. Bacteria were grown in the presence of phenamil (100 mM), an inhibitor of the sodium-dependent flagellar motor; controls received solvent alone (DMSO). **(B)** PhlyP was quantified in culture fluids (CFS) of *Pdd* strains indicated in the legend of **(A)** by dot blot immune assay. Shown are mean values ± SEM (*n* = 3); asterisk indicates *p* ≤ 0.05 (Student’s *t*-test).

Because disruption of *cheA* affected chemotactic motility on soft-agar (Figure 2), we wished to know whether interference with bacterial motility *per se* would also impact on the production of PhlyP. To this end we employed phenamil, an inhibitor of the Na^+^-channel of the flagellar motor (Atsumi et al., 1990). Phenamil reduced motility as expected (Supplementary Figure S2). This compound did not affect overall hemolysin activity in WT *Pdd*. Hemolytic activity in CFS of AR119, which produce PhlyP as the only major hemolysin, appeared to be reduced, but the effect did not reach statistical significance at *n* = 3 (Figure 5A). However, dot blot analysis revealed that production of PhlyP is inhibited in the presence of phenamil (Figure 5B). Therefore, toxin-production may depend on motility. Alternatively, signaling pathways affected by phenamil might be required to produce PhlyP.

### Influx of PI Into Epithelial Cells Infected With *Pdd* Is Reduced by Disruption of *cheA*

**FIGURE 6:**
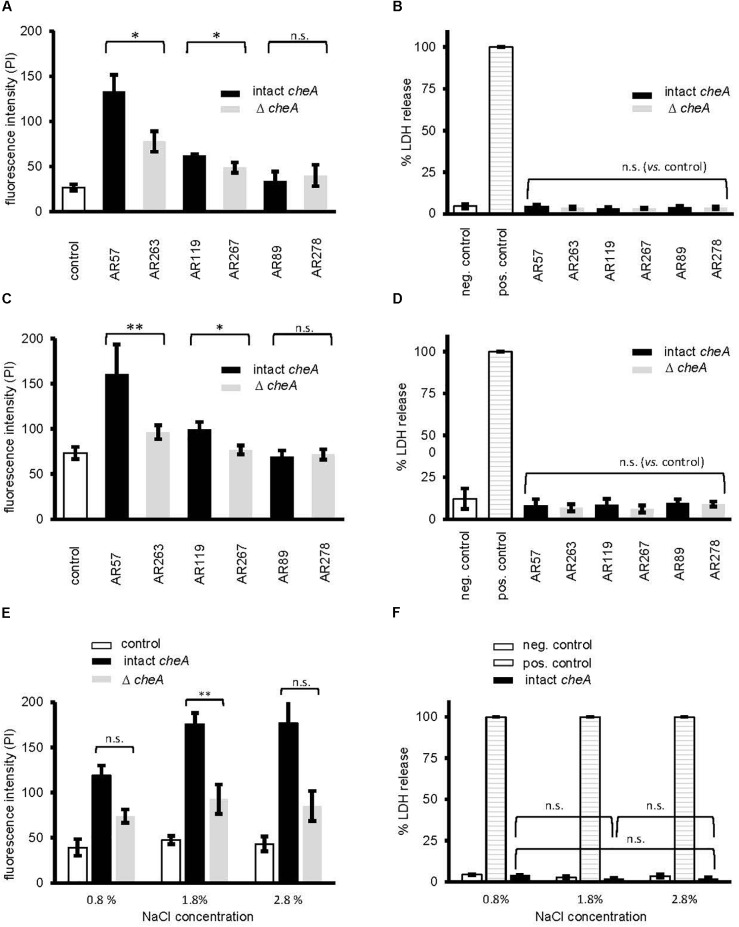
Disruption of cheA modulates permeabilization of keratinocytes by *Pdd*. **(A)** membrane permeabilization for propidium iodide was measured in HaCaT cells (4 × 10^5^ cells/well) co-cultured (MOI 1:30, for 15 min at 37°C) with *Pdd* strains, that produce all of the three major hemolysins (PhlyP, PhlyC, Dly), only PhlyP, or none of these toxins, or the corresponding *cheA* disruption mutants. Cells were washed with PBS, detached, spun down and re-suspended in PBS with EDTA (1 mM). Subsequently, cells were stained with PI (50 μg/ml) and analyzed by flow cytometry. Column heights indicate fluorescence intensity (mean channel), mean ± SEM (*n* = 3), asterisk indicates *p* ≤ 0.05 (Student’s *t*-test). **(B,D,F)** Lactate dehydrogenase release was measured using CytotoxOne-assay (Promega); positive control: Triton 1%; negative control: PBS **(B)** Release of lactate dehydrogenase (LDH) was measured in cultures of HaCaT cells (2 × 10^4^) infected (MOI 1:30, 15 min at 37°C) with *Pdd* strains as in **(A)**. **(C)** Membrane permeabilization for propidium iodide was assessed in Normal Human Epidermal Keratinocytes (NHEK), 2 × 10^5^ cells, co-cultured (MOI 1:30, 15 min at 37°C) with *Pdd* strains, by measuring PI-influx as in **(A)**. **(D)** Release of lactate dehydrogenase (LDH) from NHEK (2 × 10^4^) following co-culture (MOI 1:30, 15 min at 37°C) with *Pdd* strains was measured as in **(B)**. **(E)** The effect of salinity on membrane permeabilization for PI, of HaCaT cells, 4 × 10^5^, co-cultured (MOI 1:30, 15 min at 37°C) with the WT *Pdd* strain, or the corresponding strain with disruption of *cheA* assessed as in **(A)**.

Next, we investigated whether disruption of *cheA* impairs the ability of *Pdd* to cause toxin-dependent permeabilization of the target cell membrane. HaCaT cells were co-cultured for 15 min with *Pdd* strains, briefly incubated with PI and staining of cells was measured by flow cytometry (Figure 6A). Infection with WT *Pdd* leads to significant toxin-dependent influx of PI into HaCaT cells. Similar results were obtained with CDC2227-81, an isolate from a human patient (Kreger, 1984) (Supplementary Figures S3A–C and Supplementary Table S1). Disruption of *cheA* in the WT *Pdd* background mitigated influx of PI (Figure 6A). Notably, cells remained impermeable for LDH within the time frame of the experiment (Figure 6B and Supplementary Figure S3B); and the basic response pattern observed with HaCaT cells was recapitulated with NHEK (Normal Human Epidermal Keratinocytes) (Figures 6C,D). It is concluded that early permeabilization of keratinocytes by *Pdd* is due to formation of small pores by phobalysins, but does not yet involve membrane rupture, or membrane damage by programmed cell death routines triggered by PFT (e.g., Gonzalez-Juarbe et al., 2015; Kitur et al., 2015). Consistently, inhibitors of necroptosis (necrostatin-1), or pyroptosis (zvad-fmk) did not significantly alter influx of PI under these conditions (Supplementary Figure S4). In another set of experiments we investigated whether salinity impacts permeabilization of the infected cells. When cells were infected with WT *Pdd* at an intermediate NaCl concentration (1.8%) the influx of PI was higher than at near-physiological salinity (0.8%), but it was significantly less increased when cells were infected with the corresponding *cheA*-disrupted strain, suggesting that membrane permeabilization was not a consequence of non-physiologic salinity. Instead, the data fuel the concept that toxin genes and chemotaxis genes jointly promote membrane damage by *Pdd* in a salinity dependent way.

### Moderate Salinity, Intact *che*A and PhlyP Promote Association of *Pdd* With Epithelial Cells

**FIGURE 7:**
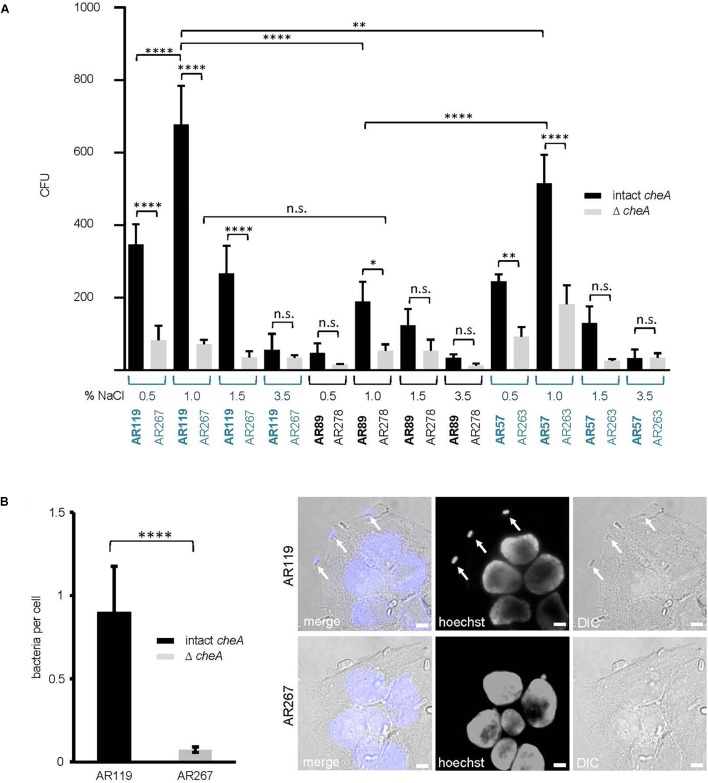
*cheA* and PhlyP jointly promote association of *Pdd* with HaCaT cells. **(A)** HaCaT cells were co-cultured with indicated *Pdd* strains previously grown overnight in LB containing different salt concentration ranging from 0.5 to 3.5% NaCl. The next day, bacteria were diluted and grown to OD_600_ = 0.4. Cells and bacteria were co-cultured for 15 min at 37°C. Subsequently, cultures were carefully washed twice. Cells with adherent bacteria were harvested and re-suspended in PBS. Dilutions of each sample were plated onto LB-agar-plates and incubated overnight at 25°C. Shown are colony counts, mean values ± SEM (*n* ≥ 5); four asterisks: highly significant differences (*p* ≤ 0.0001) in multiple comparison analysis using ANOVA with Tukey’s *post hoc* test; two asterisks: (*p* ≤ 0.01); n.s., non-significant. **(B)** HaCaT cells were infected with *Pdd* strains in the figure for 15 min at 22°C (MOI 1:30). Prior to infection bacteria were grown in LB medium with 1.5% NaCl to an OD_600_ = 0.4. Right panel: representative DIC images of infected cells, size bar = 5 μm. Arrows point to bacteria adhering to the cell surface. Left panel: graph showing number of bacteria per cell. Mean values ± SEM (*n* = 3 experiments, a total of 60 images per treatment, with an average of 6 cells per image). Four asterisks indicate a highly significant difference (*p* ≤ 0.0001) as assessed by Mann–Whitney test.

Because membrane attack by hemolysins is expected to release chemo-attractants, we reasoned that hemolysins and chemotaxis-regulators might cooperate to also promote association of *Pdd* with target cells, possibly depending on salinity. To approach this issue experimentally, we sought to analyze the effect of salt concentration, *cheA*(-disruption) and hemolysin production on the association of *Pdd* with HaCaT cells in context. We compared the strain producing PhlyP as the sole major hemolysin, the triple mutant (TM) strain, lacking Dly, PhlyP and PhlyC as well as WT *Pdd*, each with or without disruption of *cheA*. Analyses were performed following growth of *Pdd* strains at varying salt concentrations. Cultured HaCaT cells were infected with bacterial suspensions in PBS for 15 min and the number of bacteria adhering to these epithelial cells was determined by counting CFUs, as outlined in Section “Materials and Methods.” The data is summarized in Figure 7A. Provided *cheA* was intact, overall CFU counts were 2 to 3-fold higher with the hemolysin producing strains as compared to the TM strains, in keeping with previous results obtained with tissue culture media (Rivas et al., 2015b). Second, the highest CFU counts were obtained with PhlyP-only-producing *Pdd* kept in 1% NaCl prior to infection of HaCaT cells, whereas the lowest counts were invariably found with bacteria pre-cultured at high salt concentration (3.5%). Third, disruption of *cheA* led to a strong decrease of CFU with hemolysin producing *Pdd* strains grown at 1% NaCl, but did not significantly decrease the already low adherence of bacteria grown in 3.5% NaCl. Differential interference contrast microscopy confirmed that disruption of *cheA* markedly reduced the number of PhlyP-producing *Pdd* associating with epithelial cells (Figure 7B).

Using the same conditions as in the experiment of Figure 7A, we investigated the effect of small molecular weight inhibitors of various signaling pathways on the association of WT *Pdd* with HaCaT cells. In line with results obtained under cell culture conditions (Rivas et al., 2015b), a significant reduction of CFU was found in the presence of dynasore, a selective inhibitor of the large 100 kDa GTPase dynamin (Itoh et al., 2005; Kruchten and McNiven, 2006); the chelator EDTA exerted a similar effect. Notably, these compounds did not significantly reduce association of the TM strain with HaCaT cells (Table 3). This supports the notion that also in nutrient deprived media the toxin-dependent increase of adhesion is in part due to the modification of adhesive properties of these cells.

**FIGURE 8:**
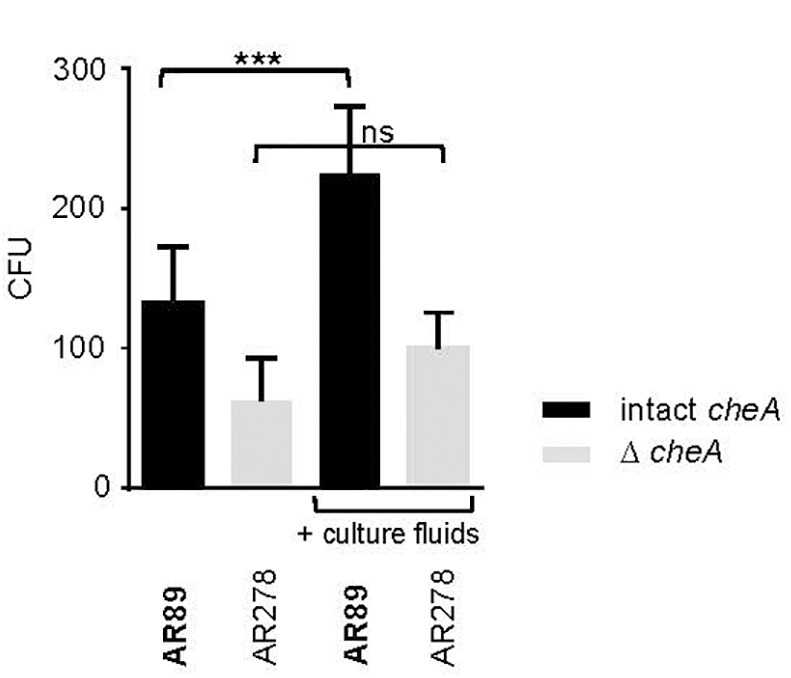
Exotoxins increase adhesion of non-toxigenic *Pdd*. HaCaT cells were infected with toxin deficient *Pdd* strains with, or w/o disruption of *cheA*, in the absence or presence of CFS derived from WT *Pdd* as a source of hemolysins. Bacteria associated with epithelial cells were determined by the CFU-based method. Data show mean values (*n* = 5); error bars indicate SEM; three asterisks indicate *p* ≤ 0.001 (one way ANOVA).

In spite of this, adhesion of *Pdd* appeared to be also regulated by *hlyA_pl_* and *cheA* (Figure 7), apparently in a cooperative way. This suggests the possibility that chemotaxis is involved. To address this question, we investigated whether the presence of exogenous toxins could enhance the association of toxin-deficient *Pdd* (TM) with HaCaT cells in a *cheA* dependent way. This approach ruled out that any effect of *cheA* on adhesion was due to its effect on the production of PhlyP. Statistically significant enhancement by exogenous hemolysins of bacterial target cell association was only seen when *cheA* was intact (Figure 8). Therefore, it is possible that hemolysin-dependent association of *Pdd* with HaCaT cells involves bacterial chemotaxis, but further experimentation is needed to clarify this point.

## Discussion

### Regulation of a Pore Forming Toxin by the Chemotaxis Apparatus

The principal novelty of the present study is the discovery of a regulatory link between a bacterial cytolytic activity and the molecular chemotaxis machinery: disruption of *cheA* or treatment with phenamil, an inhibitor of the sodium channel of the flagellar motor, both reduced motility and production of PhlyP. This suggests that motility, or the machinery regulating chemotactic motility, impacts production of this pore forming toxin. One straightforward explanation would be that *cheA* directly regulates PhlyP production. This is reasonable, first, because CheA belongs to a family of proteins that control gene expression (Stock et al., 1988). Second, *rstB*, encoding a protein of another trans-membrane bi-component system has been recently implicated in regulating hemolysin expression in *Vibrio alginolyticus* and *Pdd* (Terceti et al., 2017; Huang et al., 2018). In *Pdd*, deletion of *rstB* abolished secretion of PhlyP, PhlyC and Dly. In contrast, disruption of *cheA* appears to selectively reduce production of PhlyP; production of related PhlyC is less affected. Consistently, the promoters of the two phobalysins share little similarity (Rivas et al., 2013a). Different down-stream phosphorylation-targets of CheA and RstB could also account for the different patterns of regulation. Alternatively, rather than suppressing gene expression, disruption of *cheA*, or treatment with phenamil could act further downstream in the process of PhlyP production. The selectivity of *cheA*-dependent regulation for PhlyP suggests that *cheA*-disruption does not affect type II secretion, which has been implicated in the secretion of PhlyP, PhlyC and Dly (Rivas et al., 2015a). Conspicuously, however, the type II secretion apparatus of *V. cholerae* localizes to the same bacterial pole where its polar flagella is inserted. Therefore, it is conceivable that a *cheA*-dependent subcellular transport mechanism is co-used to target flagellar components and PhlyP to the bacterial pole. Finally, the possibility must not be dismissed that regulation of PhlyP-production by elements of the chemotaxis machinery might occur at multiple levels.

### PhlyP-Dependent Bacterial Adhesion: *cheA* Dependent vs. *cheA* Independent Effects

Our current results reveal that, mechanistically, phobalysin-dependent association of *Pdd* with epithelial cells is a composite effect: first is a toxin dependent increase of the adhesive properties of target cells, an effect that is sensitive to dynasore or EDTA (this work and Rivas et al., 2015b). Second, *hlyA*_pl_ and *cheA* cooperatively enhance the association of *Pdd* with epithelial cells at intermediate to low salinity (this work). It is possible that cooperativity is largely due to *cheA*-dependent regulation of PhlyP production, but it is equally possible that chemotaxis itself is involved. Although speculative, a model of exotoxin-guided chemotactic orientation (ECHO) would accommodate the *cheA*-dependent enhancement by exogenous PhlyP of the adhesion of non-toxigenic *Pdd* (AR89): solute gradients emanating from damaged cells, and blood seeping from the wound would provide a cue for directional, *cheA*-dependent migration of *Pdd* toward its host. Solutes may be released by mechanical wounding and/or through membrane damage by bacterial toxins (Zitzer et al., 1997). Mammalian cells under attack by a pore forming toxin release potassium, which is a strong chemo-attractant, as already described in pioneering studies (Pfeffer, 1888). Chemo-attractants definitively involved in *cheA*/PhlyP-dependent target cell association remain to be identified. The speed of both PhlyP secretion by bacteria (Rivas et al., 2015b), and PhlyP-induced release of potassium from target cells (von Hoven et al., 2017) could support the ECHO-scenario. In this context, even in a turbulent marine environment, chemical gradients emanating from lysing cells (protozoa) are able to attract marine bacteria (Fenchel, 2002). Also, autocrine, *cheA*-dependent signaling could mediate hemolysin-dependent association of *Pdd* with epithelial cells (Doganer et al., 2016); and involvement of hormones involved in quorum sensing of marine bacteria has yet to be excluded (Cude and Buchan, 2013).

### Transition of *Pdd* From an Environmental Mode to an Infection Mode

We had previously found that association of WT *Pdd* with human epithelial cells (HaCaT) was not significantly affected by a disruption of *cheA*. Obviously, the use of nutrient replete tissue culture media concealed the moderate *cheA*-dependent response of the WT strain. The importance of salinity is illustrated by slightly different optima of salinity for swimming (1.5–2% NaCl), as compared to the production of plasmid-encoded toxins, PhlyP and Dly, which was highest at 1% NaCl. Upon contamination of a wound, salt water will mix with wound secretion, resulting in a milieu of intermediate salt concentration. This appears to be a favorable condition for adhesion of *Pdd* and damage to epithelial cells. The salt-dependent changes observed with *Pdd* may thus reflect a transition from an environmental mode to an infection mode. Many environmental factors are likely involved in regulating this transition, and additional bacterial functions may be affected. Microfluidics-based assays might be helpful for simulating certain environmental conditions (Lambert et al., 2017). Ultimately, it will be necessary to study the relevance of our present findings *in vivo*.

### Emerging Links Between Chemotaxis, Cytotoxicity, and Adhesion

Although different model systems and different experimental conditions obviate direct comparison, findings of other groups are reminiscent of some of the observations made here with *Pdd*. For instance, the release of PhlyP from *Pdd* grown at intermediate salt concentrations was higher as compared to high salinity conditions; similar findings have been reported for *V. vulnificus* toxin (Lee et al., 2000). Also, swimming of *Pdd* in 1.5% NaCl was stronger than in 3.5% NaCl, similar to the chemotactic response of *V. anguillarum* (Larsen et al., 2004). Moreover, a role of various regulators of chemotaxis for adhesion has been reported previously: *cheA* has been implicated in the attachment of *Marinobacter adhaerens* HP15 to diatom surfaces (Sonnenschein et al., 2012); other regulators of chemotaxis were found to impact adhesion of *V. alginolyticus* to a mucus layer (Huang et al., 2017). To sum, functional connections between chemotaxis and adhesion have been reported, and a role of hemolysins for regulating bacterial association with target cells has recently emerged (Krawczyk-Balska and Bielecki, 2005; Lucas et al., 2010; Vadia et al., 2011; Seitz et al., 2013; Rivas et al., 2015b). However, we are aware of only one report discussing in context elements of the chemotaxis apparatus and the protein machinery involved in hemolysin action: *V. cholerae* HlyB, a membrane associated protein involved in the secretion of hemolysins is a member of the chemotaxis receptor gene family (Jeffery and Koshland, 1993). Along this line, the present work uncovers a salinity-dependent regulatory link between the chemotaxis apparatus and a cytotoxic activity, which jointly increase the association of bacteria with target cells.

## Author Contributions

GH, MH, CO, and AR conceived of the study. GH, MH, and AR wrote the manuscript, designed experiments, analyzed data, and interpreted them. CO interpreted data. CN did most of the experimental work and contributed to data analysis and interpretation. GH, AR, SS, AV, and MM performed experiments and acquired data. All authors critically revised the manuscript and approved the final version.

## Conflict of Interest Statement

The authors declare that the research was conducted in the absence of any commercial or financial relationships that could be construed as a potential conflict of interest.

## Supplementary Material

FIGURE S1 AR89 and AR278 are non-hemolytic and complementation of AR89 with hlyA_pl_ (AR272) restores hemolysis. **(A,B)** Culture fluids (CFS) of indicated *Pdd* strains grown at different salt concentrations were diluted and hemolytic activity was measured. Shown are mean values ± SEM; *n* = 3. **(B)** Strains as in **(A)** were incubated on soft agar plates with indicated NaCl concentrations, and diameter of swim rings was measured the next day. Data shown are mean values ± SEM (*n* = 3).

FIGURE S2 Phenamil inhibits motility of *Pdd*. **(A)** Bacteria were pre-incubated for 15 min in the presence of phenamil (20 μM), or solvent alone (DMSO). Subsequently, bacteria were washed and a capillary assay was used to document differential motility; growth was not affected by phenamil. Shown are mean values ± SEM (*n* = 5).

FIGURE S3 Cytotoxicity of *Pdd* isolate CDC 2227.81. **(A)** To assess membrane permeabilization for PI in HaCaT cells, 4 × 10^5^ cells were co-cultured (MOI 1:30, 20, 30 or 40 min at 37°C) with CDC 2227.81 a human isolate of *Pdd*. Cells were washed with PBS, detached, spun down and re-suspended in PBS with EDTA (1 mM). Subsequently, cells were stained with PI (50 μg/ml) and analyzed by flow cytometry. Column hight indicates fluorescence intensity (mean channel), mean ± SEM (*n* ≥ 4). **(B)** Release of lactate dehydrogenase (LDH) was measured in cultures of HaCaT cells (2 × 10^4^) infected with CDC 2227.81 as in **(A)**, (MOI 1:30, 20, 30 or 40 min at 37°C). **(C)** Hemolysis was measured with CFS of CDC 2227.81 and normalized as detailed in the Methods section. Shown are mean values ± SEM (*n* = 3).

FIGURE S4 Inhibitors of necroptosis or pyroptosis do not alter rapid permeabilization of HaCaT by *Pdd*. **(A)** The effect of necrostatin-1 (Santa Cruz Biotechnology, SC 200142), or z-VAD-FMK (Selleckchem, Cat.-N° S7023), each at 100 μM, on membrane permeabilization for PI was measured in HaCaT cells (4 × 10^5^) cells co-cultured (MOI 1:30, 15 min at 37°C) with strain AR57 or AR119. Control cells received solvent alone (DMSO). Mean values ± SEM (*n* = 3).

TABLE S1 Additional strains used for supplemental data.
