# Supplementary figures and images for: Cytotoxin- and Chemotaxis-Genes Cooperate to Promote Adhesion of Photobacterium damselae subsp. damselae
Source: Front Microbiol. 2018 Dec 13;9:2996. doi: 10.3389/fmicb.2018.02996 (PMC6300472; doi:10.3389/fmicb.2018.02996)

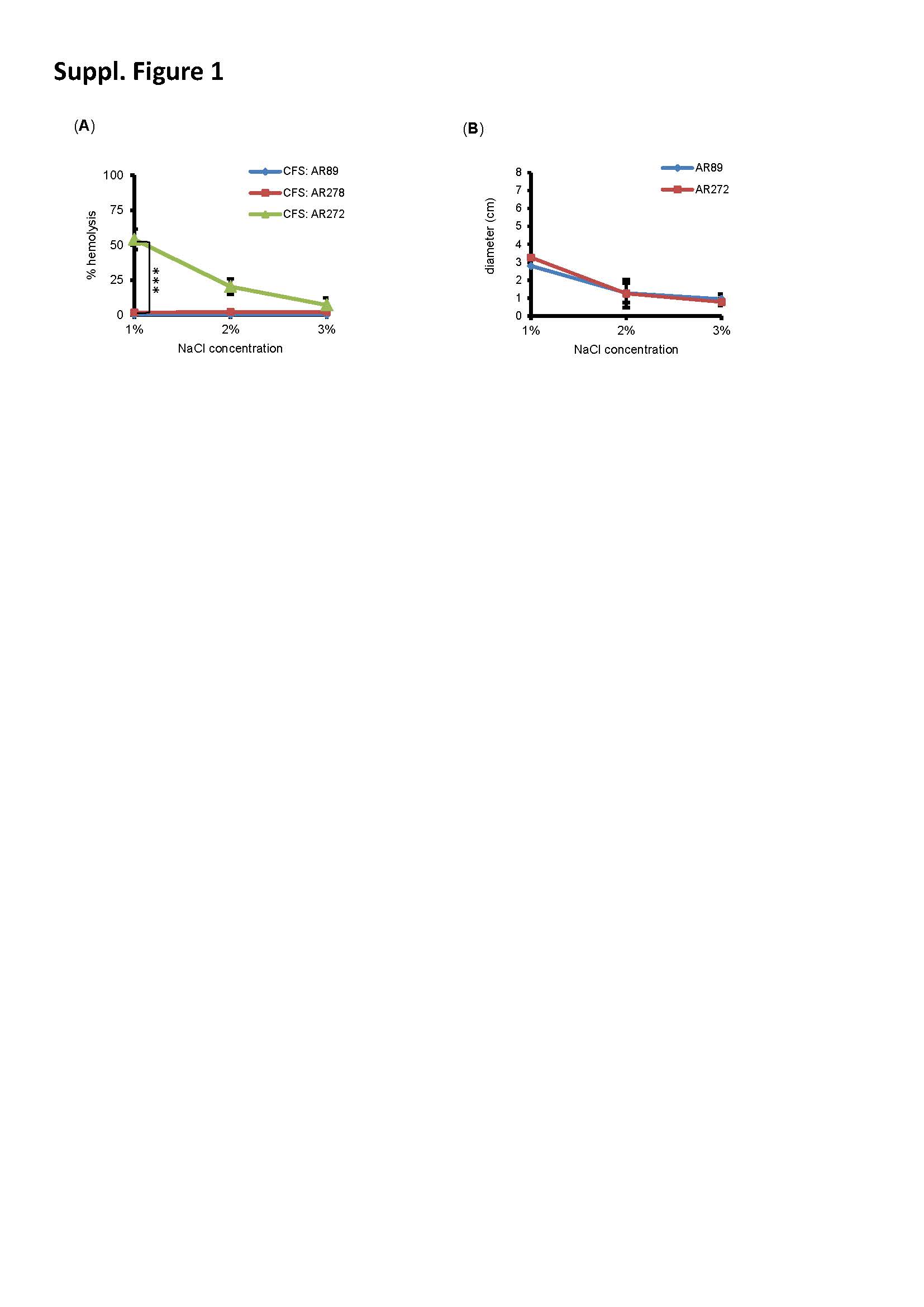

Supplement: FIGURE S1 — AR89 and AR278 are non-hemolytic and complementation of AR89 with hlyApl (AR272) restores hemolysis. (A,B) Culture fluids (CFS) of indicated Pdd strains grown at different salt concentrations were diluted and hemolytic activity was measured. Shown are mean values ± SEM; n = 3. (B) Strains as in (A) were incubated on soft agar plates with indicated NaCl concentrations, and diameter of swim rings was measured the next day. Data shown are mean values ± SEM (n = 3). [file Image_1.JPEG]

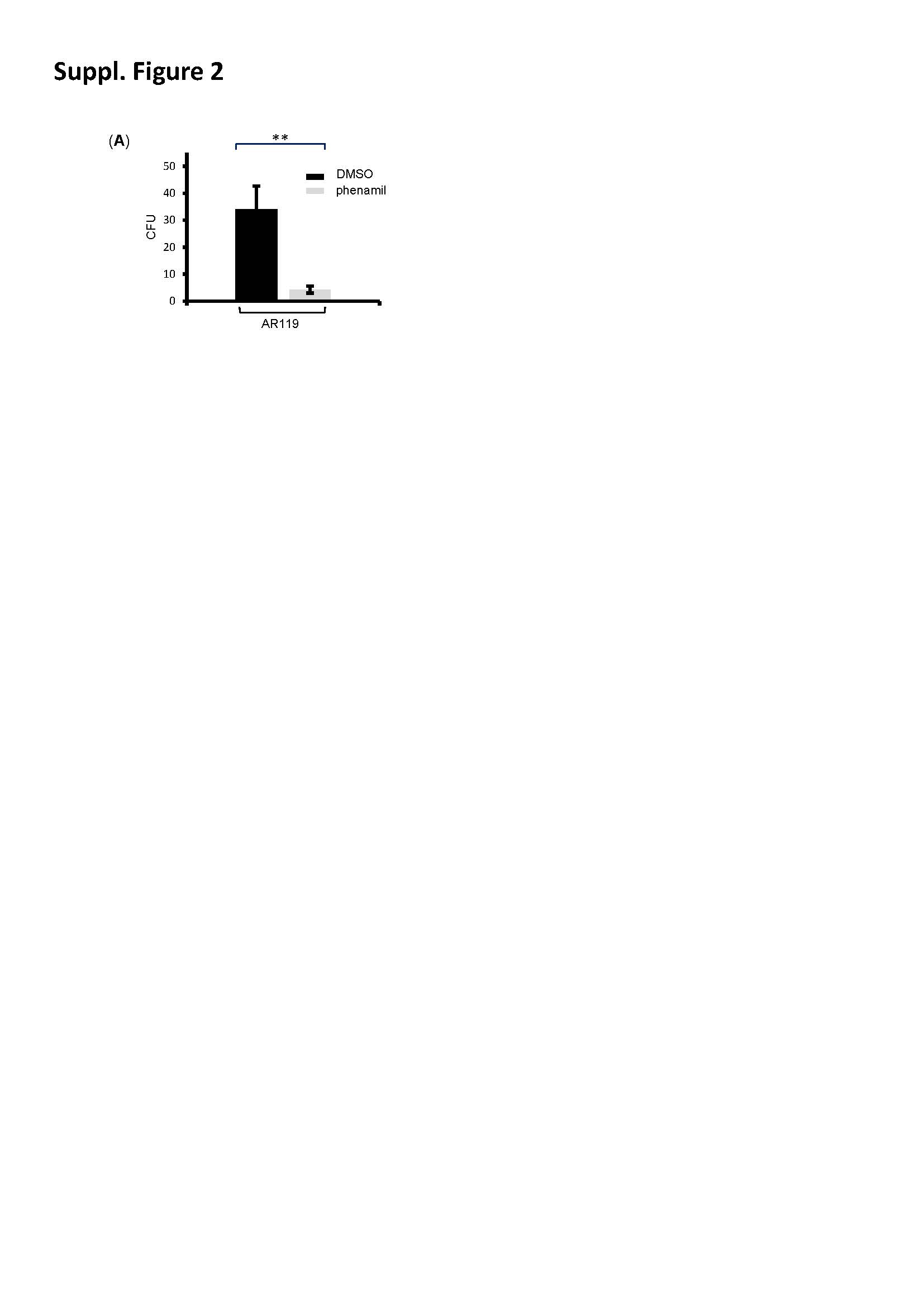

Supplement: FIGURE S2 — Phenamil inhibits motility of Pdd. (A) Bacteria were pre-incubated for 15 min in the presence of phenamil (20 μM), or solvent alone (DMSO). Subsequently, bacteria were washed and a capillary assay was used to document differential motility; growth was not affected by phenamil. Shown are mean values ± SEM (n = 5). [file Image_2.JPEG]

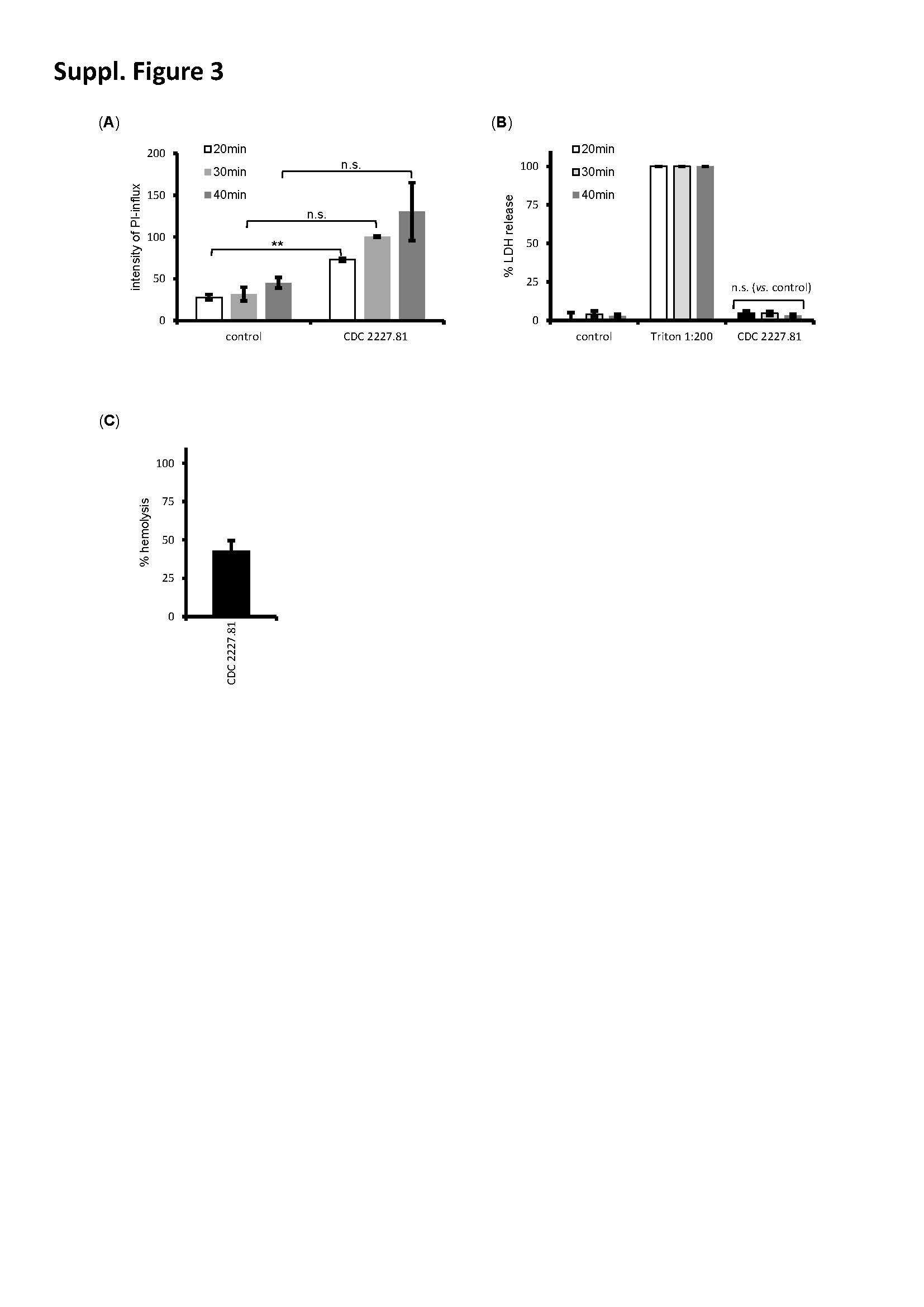

Supplement: FIGURE S3 — Cytotoxicity of Pdd isolate CDC 2227.81. (A) To assess membrane permeabilization for PI in HaCaT cells, 4 × 10^5^ cells were co-cultured (MOI 1:30, 20, 30 or 40 min at 37°C) with CDC 2227.81 a human isolate of Pdd. Cells were washed with PBS, detached, spun down and re-suspended in PBS with EDTA (1 mM). Subsequently, cells were stained with PI (50 μg/ml) and analyzed by flow cytometry. Column hight indicates fluorescence intensity (mean channel), mean ± SEM (n ≥ 4). (B) Release of lactate dehydrogenase (LDH) was measured in cultures of HaCaT cells (2 × 10^4^) infected with CDC 2227.81 as in (A), (MOI 1:30, 20, 30 or 40 min at 37°C). (C) Hemolysis was measured with CFS of CDC 2227.81 and normalized as detailed in the Methods section. Shown are mean values ± SEM (n = 3). [file Image_3.JPEG]

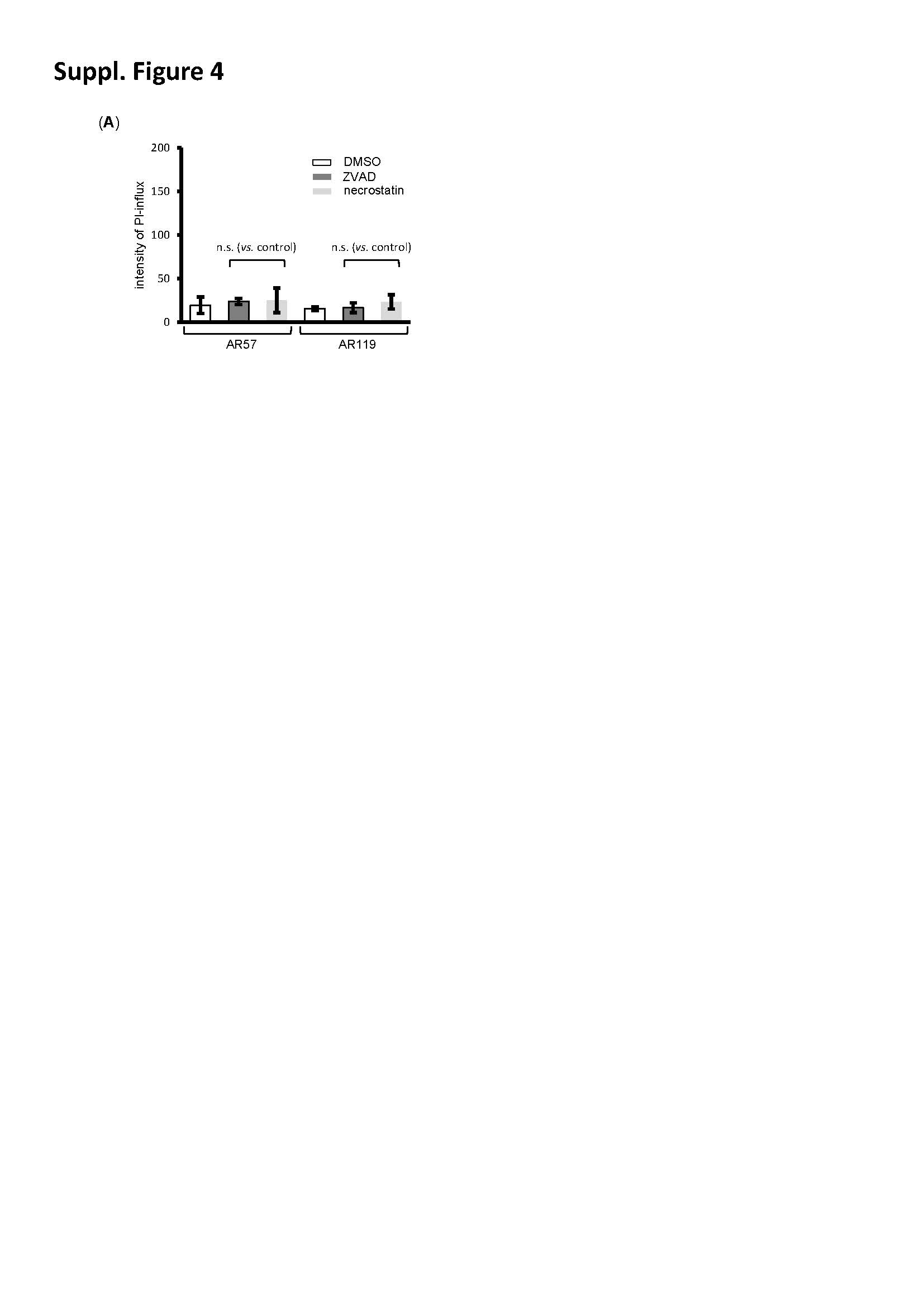

Supplement: FIGURE S4 — Inhibitors of necroptosis or pyroptosis do not alter rapid permeabilization of HaCaT by Pdd. (A) The effect of necrostatin-1 (Santa Cruz Biotechnology, SC 200142), or z-VAD-FMK (Selleckchem, Cat.-N° S7023), each at 100 μM, on membrane permeabilization for PI was measured in HaCaT cells (4 × 10^5^) cells co-cultured (MOI 1:30, 15 min at 37°C) with strain AR57 or AR119. Control cells received solvent alone (DMSO). Mean values ± SEM (n = 3). [file Image_4.JPEG]
