# Supplementary material for: Cytotoxin- and Chemotaxis-Genes Cooperate to Promote Adhesion of Photobacterium damselae subsp. damselae
Source: Front Microbiol. 2018 Dec 13;9:2996. doi: 10.3389/fmicb.2018.02996 (PMC6300472; doi:10.3389/fmicb.2018.02996)
Supplement: TABLE S1 — Additional strains used for supplemental data. [file Table_1.pdf]

P. damselae subsp. *damselae*	disruption in *cheA* gene	Reference	toxins produced by the strains	Description
AR272	-	this study	PhlyP	AR57 with in-frame deletion of $hlyA_{pl}$, $hlyA_{ch}$ and *dly* genes complemented with $hlyA_{pl}$
CDC 2227-81	-	Kreger et al. 1984	PhlyP, PhlyC, Dly	human isolate, Massachusetts United States 1981; Centers for Disease Control

Table S1: Additional strains used for supplemental data
